# Phenotyping grapevine red blotch virus and grapevine leafroll-associated viruses before and after symptom expression through machine-learning analysis of hyperspectral images

**DOI:** 10.3389/fpls.2023.1117869

**Published:** 2023-03-10

**Authors:** Erica Sawyer, Eve Laroche-Pinel, Madison Flasco, Monica L. Cooper, Benjamin Corrales, Marc Fuchs, Luca Brillante

**Affiliations:** ^1^ Viticulture & Enology Research Center, California State University Fresno, Fresno, CA, United States; ^2^ Department of Mathematics, California State University Fresno, Fresno, CA, United States; ^3^ Department of Viticulture & Enology, California State University Fresno, Fresno, CA, United States; ^4^ Plant Pathology and Plant-Microbe Biology, Cornell University, Geneva, NY, United States; ^5^ University of California, Agriculture & Natural Resources, Napa, CA, United States

**Keywords:** phenomics, spectroscopy, *Vitis vinifera* L., disease detection, deep-learning, convolutional neural network, random forest

## Abstract

**Introduction:**

Grapevine leafroll-associated viruses (GLRaVs) and grapevine red blotch virus (GRBV) cause substantial economic losses and concern to North America’s grape and wine industries. Fast and accurate identification of these two groups of viruses is key to informing disease management strategies and limiting their spread by insect vectors in the vineyard. Hyperspectral imaging offers new opportunities for virus disease scouting.

**Methods:**

Here we used two machine learning methods, i.e., Random Forest (RF) and 3D-Convolutional Neural Network (CNN), to identify and distinguish leaves from red blotch-infected vines, leafroll-infected vines, and vines co-infected with both viruses using spatiospectral information in the visible domain (510-710nm). We captured hyperspectral images of about 500 leaves from 250 vines at two sampling times during the growing season (a pre-symptomatic stage at veraison and a symptomatic stage at mid-ripening). Concurrently, viral infections were determined in leaf petioles by polymerase chain reaction (PCR) based assays using virus-specific primers and by visual assessment of disease symptoms.

**Results:**

When binarily classifying infected vs. non-infected leaves, the CNN model reaches an overall maximum accuracy of 87% versus 82.8% for the RF model. Using the symptomatic dataset lowers the rate of false negatives. Based on a multiclass categorization of leaves, the CNN and RF models had a maximum accuracy of 77.7% and 76.9% (averaged across both healthy and infected leaf categories). Both CNN and RF outperformed visual assessment of symptoms by experts when using RGB segmented images. Interpretation of the RF data showed that the most important wavelengths were in the green, orange, and red subregions.

**Discussion:**

While differentiation between plants co-infected with GLRaVs and GRBV proved to be relatively challenging, both models showed promising accuracies across infection categories.

## 1 Introduction

California vineyards are affected by two major viral diseases with similar symptoms and consequences on grape quality and quantity: leafroll and red blotch (Sudarshana et al., 2015). Six grapevine leafroll-associated viruses (GLRaVs) are associated with leafroll disease, among which GLRaV-3 is predominant (Naidu et al., 2015). These viruses affect fruit ripening, decrease grape quality, and reduce yield by up to 68% (Atallah et al., 2012). Grapevine red blotch virus (GRBV) causes red blotch disease (Yepes et al., 2018). This virus slows down and can stop the accumulation of sugars and phenolic compounds (Ricketts et al., 2017; Martínez-Lüscher et al., 2019). Both viral diseases show similar foliar symptoms of leaf reddening on red wine grape cultivars (Sudarshana et al., 2015). Without any control measures, both diseases can cause economic losses of up to $226,405/ha for leafroll (Ricketts et al., 2015) and up to $68,548/ha for red blotch (Ricketts et al., 2017) over the approximate 25-year lifetime of a vineyard.

To date, scouting and removing symptomatic vines and replanting them with healthy ones (i.e., roguing) is the principal strategy employed by growers to limit the secondary spread of both viruses by their insect vectors in diseased vineyards. Most GLRaVs are transmitted by mealybugs and soft-scale insects (Naidu et al., 2015), while GRBV is transmitted by the three-cornered alfalfa hopper (Flasco et al., 2021). Roguing infected vines is efficient against these diseases, but diagnosing infected plants based on visual symptoms is time-consuming. It is also impractical as symptoms are only expressed late in the season when growers are busy with harvest operations and have limited time for additional tasks. Moreover, expertise is required to precisely identify infected plants and avoid misdiagnosis, as both diseases can be confused with other pathological, nutritional, and physiological issues (Sudarshana et al., 2015). The use of molecular diagnostic methods is the golden standard for assessing viral infections in vines. Unfortunately, these assays are costly and time-consuming, so a census approach to testing vines is not feasible (testing each vine one-by-one). A more automated way to quickly detect and diagnose viral diseases would be undeniably beneficial to vineyard managers.

Spectroscopy is a set of powerful tools which can help identify plants infected with diseases that affect biochemical and biophysical plant properties, changing their optical signatures (Knipling, 1970; Croft and Chen, 2018). These sensing techniques can also be applied remotely, thus offering the ability for rapid-scale identification over large areas. Hyperspectral imaging spectrometry is a very effective remote sensing tool, as individual wavelength information can be obtained over large regions of the electromagnetic spectrum while maintaining spatial information. Consequently, there has been a rapid increase in research activity in this field in recent years (Terentev et al., 2022). In grapevine, several studies have used hyperspectral data to identify pests and diseases such as phylloxera (Vanegas et al., 2018), leaf stripe disease (Junges et al., 2018), flavescence dorée (AL-Saddik et al., 2017), leafroll (MacDonald et al., 2016; Sinha et al., 2019; Bendel et al., 2020; Gao et al., 2020) and red blotch (Mehrubeoglu et al., 2016). To our knowledge, no study on hyperspectral imaging methodologies has attempted to distinguish between the leafroll and red blotch virus infection.

Deep learning methods, such as the Convolutional Neural Network (CNN) (Lecun and Bengio, 1995), are particularly well-suited for disease detection on images, as they can detect underlying structures and spatial patterns (Lee et al., 2015; Grinblat et al., 2016; Kerkech et al., 2020). However, few studies on detecting grapevine diseases used deep learning models (Hruska et al., 2018; Nguyen et al., 2021). To the best of our knowledge, no prior studies have compared visual assessment to machine-learning detection of viral diseases in grapevines. Our study fills this gap by comparing two machine learning models, CNN and RF, to detect GLRaVs, GRBV, and mixed infections of GLRaVs and GRBV from hyperspectral imagery and by contrasting predictions to molecular and visual estimates. We worked with over 400 leaf images of healthy and infected grapevines captured within the visible range (from 510nm to 710nm) at different symptomatic stages (before and after symptoms were visible). Finally, we performed an explanatory analysis to identify the most essential wavelengths to predict virus-infected vines in the visible range.

## 2 Materials and methods

**Figure 1:**
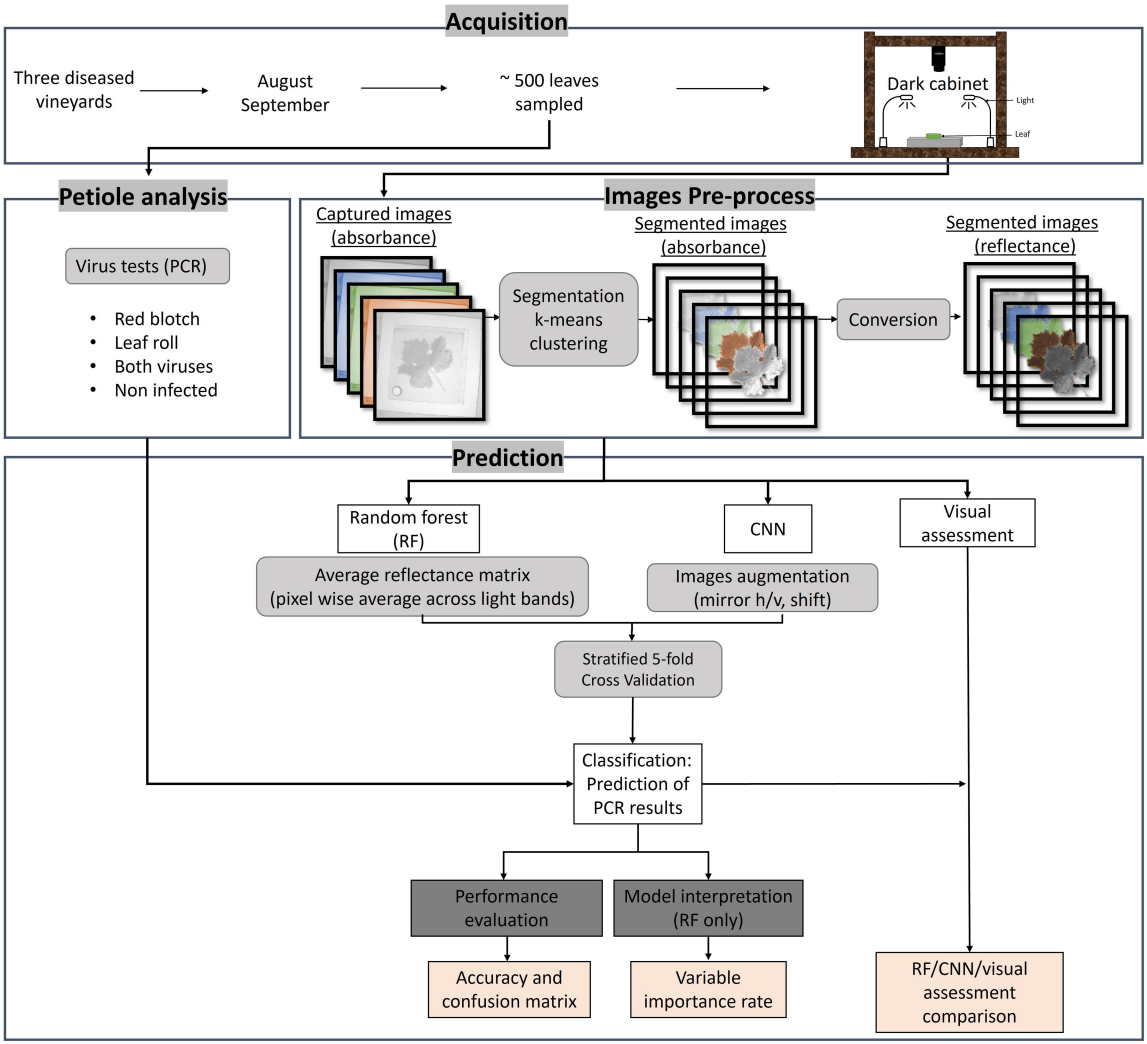
Workflow of the different methodology steps used in this study. The acquisition was done in three vineyards in August and September 2019 and 2022. About 500 leaves were sampled for PCR analysis and imaged using a hyperspectral camera in a dark cabinet. The image pre-process consisted of segmenting the leaves to extract the pure leaf signal and converting the radiance to reflectance using a white standard. The predictions of the PCR results were done using random forest (RF) and convolutional neural network (CNN). A visual assessment by experts was also done. The results were evaluated using accuracy and confusion matrices and interpreted *via* variable importance rate.

**Figure 1** summarizes the workflow used in this study. The first step consisted of collecting leaves from selected grapevines in three vineyards and testing the petioles for viruses using molecular analyses to distinguish between healthy and diseased samples. The next step consisted of imaging the leaves in the visible domain with a hyperspectral camera in a dark cabinet under controlled lighting in the laboratory. Images were then pre-processed to segment the leaves from the background, and data were transformed into reflectance values. Random Forest and CNN models were then applied to the segmented images to classify their infection status and compare diagnostic predictions of the models with the molecular test results and with a visual assessment made by experts.

### 2.1 Sampling and data collection

#### 2.1.1 Grapevine leaf sampling, image acquisition, and pre-processing

In August and September 2019 and 2022, leaves were sampled from randomly selected vines in one Cabernet franc vineyard and in two Cabernet Sauvignon vineyards, located in North and Central California (Rutherford, Fresno, Madera). Vineyards were composed of adult plants at least 10-years old and grown according to common practices for the area. All vineyards were known to exhibit leafroll- and red blotch-like symptoms. We collected four leaves per plant on the lower portion of the canopy close to the trunk. Samples were temporarily stored in a cooler and later maintained at 4°C in the laboratory. Two of the four leaves collected per vine were randomly selected for imaging in a dark cabinet under a LED light. This light did not emit in the near-infrared (Fiber-Lite Mi-LED Illuminator, Dolan-Jenner Industries USA) to remove noise related to time differences between sampling and imaging. One mega-pixel image was acquired with a Senop HIS camera using a 200ms exposure time. Bands were acquired every ~5nm from 510nm to 710nm for a total of 40 bands. In all pictures, we included a white reflectance standard (Spectralon^®^, Labsphere, USA). We separated the leaf from the background in all images using an unsupervised segmentation approach based on k-means clustering (Dhanachandra et al., 2015).

**Figure 2:**
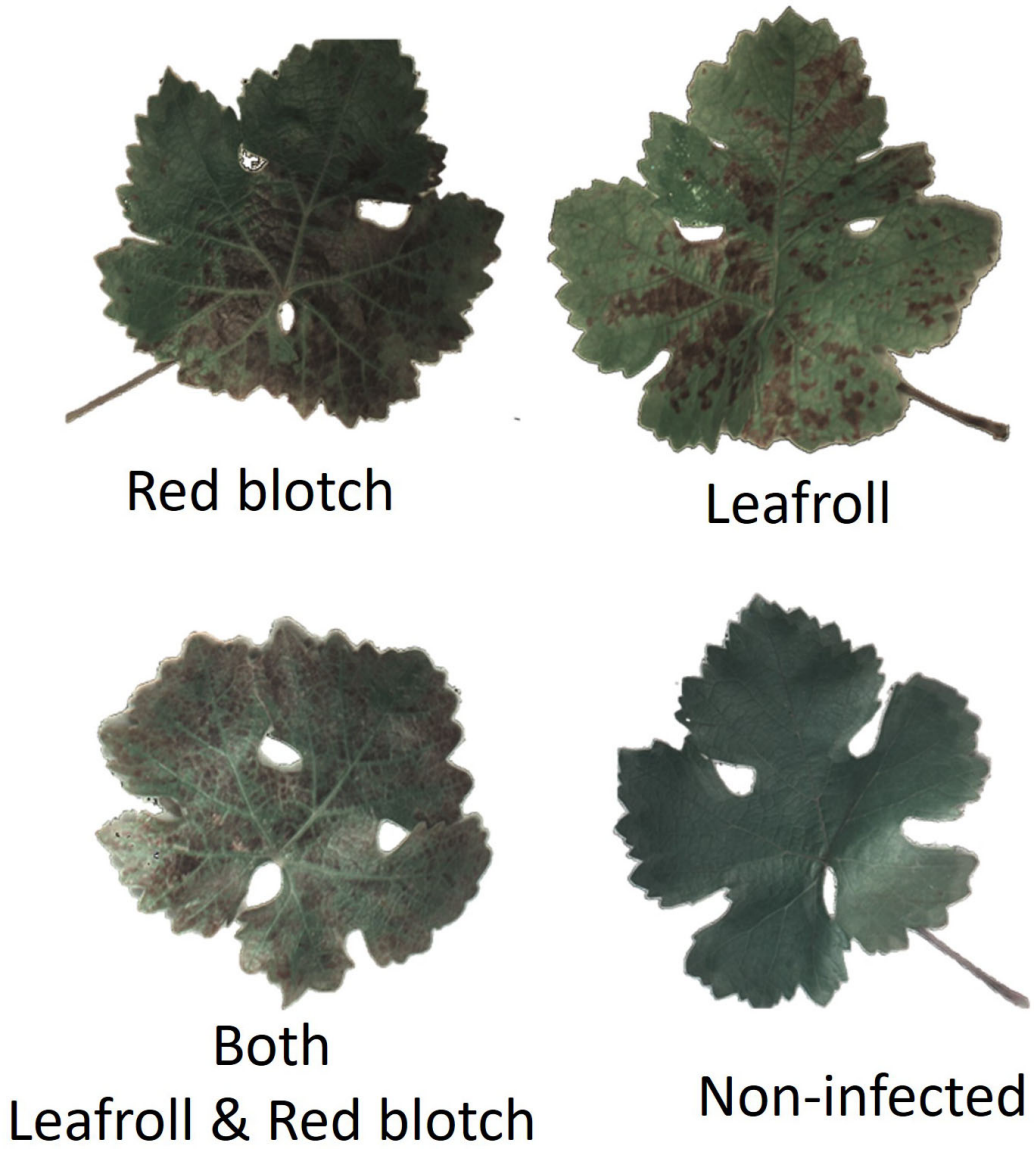
Examples of RGB segmented leaf images for each category using the reflectance at 525.3nm, 555.7nm, and 601.3nm.

Four different disease categories were identified in the image dataset: non-infected, infected with GRBV, infected with GLRaV, and co-infected with GRBV and GLRaV (**Figure 2**). Leaves from vines that tested negative for GLRaV and.or GRBV *via* PCR, although presenting reddening, were also included in the dataset (**Supplementary Figure 1**). They were classified as non-infected following PCR results.

#### 2.1.2 Assessment of virus infection

Viral infection was assessed by PCR-based analysis on the petioles of the four-leaf set collected per plant. Petioles were sliced into small pieces with sterile razor blades and used for nucleic acid isolation using the MagMAX 96 AI/ND Isolation kit (Thermo Fisher Scientific) on a King Fisher instrument. GLRaV-1, -2, -3, and -4, and GRBV were detected by RT-PCR and PCR, respectively, as previously described (Osman et al., 2007; Krenz et al., 2014).

### 2.2 Classification process

#### 2.2.1 Experts’ classification

Classification of the leaves into the four disease categories according to visual symptoms was performed on RGB segmented images by two experts in cooperation with each other and without previous information on the dataset (using 525.3nm, 555.7nm, and 601.3nm).

#### 2.2.2 Machine learning models description

Two predictive machine learning models were used in this study: Random Forest (RF) (Breiman, 2001; Parmar et al., 2019; Shaik and Srinivasan, 2019), and Convolutional Neural Network (CNN) (LeCun et al., 2015; Lu et al., 2021).

The random forest algorithm is a commonly used model for remote sensing classification (Pal, 2005; Belgiu and Drăgut, 2016). This model has found multiple applications in viticulture, such as sensing soil water (Brillante et al., 2016a), or imaging grapevine water uptake (Brillante et al., 2016b). A random forest uses decision trees in an ensemble built through a modified bagging approach. Decision trees are a rule-based model where each rule splits the dataset into more homogeneous groups with respect to the response variable. Their structure greatly varies with minor changes in the data available for modeling and ensemble methods like bagging leverage this property. In bagging, multiple trees are built using different versions of the original data set obtained through resampling techniques. In this way, each tree has a different structure and learns other aspects of the dataset; the ensemble finally outperforms the individual learners. In random forest, the perturbation process is further enhanced by the fact that the trees use only a fraction of all available predictors at each split. In this work, we tuned the number of trees in the forest and the number of predictors available at each partition using a cross-validation routine, as previously reported (Brillante et al., 2015).

A neural network is a sequence of linear and nonlinear transformations that uses training data to learn the structure of the dataset and inform optimal classifications of test data. A CNN is a specific type of neural network which consists of convolutional, normalization, nonlinear, and fully connected layers. CNNs are especially useful in the case of machine learning problems using image data, as they can isolate smaller regions of the image to reduce the amount of data that must be processed at a given time (Albawi et al., 2017). Also, the depth of CNN models allows them to adapt well to highly nonlinear data, such as the dataset being explored in this study.

**Figure 3:**
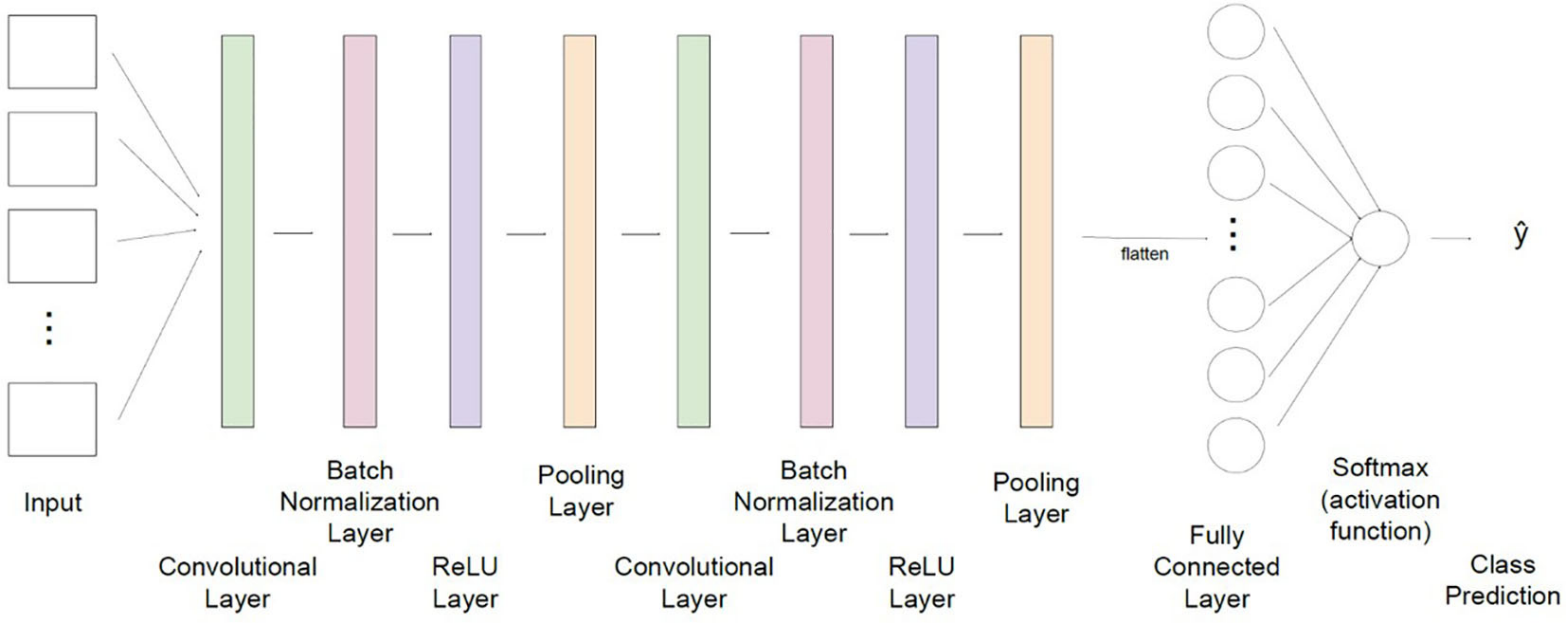
The convolutional neural network architecture used in this study for classification of grapevine disease. Input for the network consists of hyperspectral images of leaves, captured at 40 wavelengths. Data is then fed through two sequences of layers which are designed to extract significant features from each image (convolution), normalize values to minimize computational cost (batch normalization), adapt to nonlinearity of values (ReLU), and combine regional values to reduce the overall size of the dataset to be processed (pooling). A fully connected layer flattens data and updates network weights and parameters to improve prediction capability. A final activation function returns a class label which translates to the predicted virus classification (non-infected, leafroll, red blotch, and co-infected with both viruses).

The 3D-CNN architecture used in this work is shown in **Figure 3**. To predict the virus status of a given leaf, the CNN first accepts as input a hyperspectral image, which contains matrices of data representing that same image captured from 40 different wavelengths. Each matrix is passed to one of the network’s 40 channels to be simultaneously considered in recognizing significant features of the image. There are then two consecutive sequences of convolution, normalization, and rectified linear unit (ReLU) pooling layers where the model can extract important features to learn the structure of the images supplied. These sequences filter through the image provided to each input channel and first filter out values that differ significantly from the surrounding region (convolutional layer), normalize values to reduce computational cost (batch normalization layer), enhance nonlinearity of the data (ReLU layer), and shrink each region to a single value to reduce the size and decrease the likelihood of overfitting (pooling layer). Next, the output of these layers is flattened into a one-dimensional array, and a fully connected layer tunes internal parameters to adapt to the nonlinearity in these data and make a classification. The final layer of the network, namely the activation function, interprets the classification made by the fully connected layer and assigns a predicted class label, acting as the output of the CNN.

Network parameters in the fully connected layer are selected using an iterative optimization technique known as Adaptive Moment Estimation (Adam). Adam is an adaptation of Stochastic Gradient Descent (SGD), an algorithm in which network parameter values are tuned to minimize model loss by using a gradient calculated using a random subset of points in the data set. A unique feature of the Adam algorithm lies in its ability to track previous gradients which guide model parameters towards optimal values much more quickly and efficiently than similar methods, making it an appropriate option for multilayer CNNs as are used for this classification problem (Kingma and Ba, 2015). A fixed learning rate of 0.001 is used for each iteration of the Adam algorithm.

#### 2.2.3 Classification scheme

We classified the grapevine leaf samples into non-infected, leafroll-infected, red blotch-infected, and leafroll and red blotch co-infected categories according to the results from the PCR analyses and independently from the visual assessments of disease symptoms.

Due to the asymptomatic nature of the two diseases early in the growing season we considered three different datasets: one with the entire data, one with only pre-symptomatic leaves, and the last one with only symptomatic leaves (post-symptomatic). This allowed us to reliably assess the models’ ability to classify symptomatic, infected vines and compare them to the performance of asymptomatic classification.

A binary classification model was initially explored for which CNN and RF models were trained to distinguish between non-infected and infected plants. Later the models were trained in a multiclass classification scheme, for which each class was independently predicted (non-infected, infected by red blotch, infected by leafroll or co-infected by both).

#### 2.2.4 Training/testing

The prediction skills of each model were assessed using a stratified 5-fold cross-validation (CV) scheme. A k-fold cross-validation technique shuffled the dataset before partitioning it into *k* non-overlapping folds. For each unique fold, data were held once for use as a test set, while all remaining k-1 folds were combined into a training set. A model was fit on the training set and evaluated using the test data, which were previously unseen by the model. Model parameters were then cleared, and evaluation scores were recorded and averaged across all folds. This technique is less computationally costly than alternative forms of cross-validation, and yields averaged measurements with a valuable estimate for the predictive power of the model on the full dataset (James et al., 2013). This work used stratified k-fold CV, wherein folds were selected to maintain the class distribution of the full dataset. This is especially useful for cases of class imbalance, as was seen in the dataset generated in this study, where the model performance tends to be more stable from fold to fold (López et al., 2014).

The training set for the CNN model was augmented to improve network performance by providing additional training images with less predictable features. Each training image was duplicated, and the copy underwent a random combination of horizontal and vertical flips, image shifts, scaling, and rotation (where each transformation has a probability of 0.5 of being applied to a given image). This expanded and introduced additional variability in the training dataset, preventing the model from becoming familiar with leaf shape and orientation. The average signal used in the RF model was invariant to most of these transformations. Therefore, augmentation was not done for this model.

Augmentation was performed using the Albumentations package in Python (Buslaev et al., 2020). Minibatch training was utilized to reduce the computational and memory strain on the GPUs when training the CNN model. Training data was broken down into batches of 50 images each, and the model continued the tuning parameters for 30 epochs or full iterations through the dataset. There was no need to use a similar technique for the RF model, as the input into the model is a significantly smaller dataset and did not pose any computation or storage complications.

The torch.nn.CrossEntropyLoss() function in PyTorch was used to evaluate model loss for the RF and CNN methods. All experiments were run on a 20-core machine with 2 GeForce RTX 2080Ti graphic processing units (GPUs). All machine learning models were developed and evaluated using Python version 3.7.10 and including tools from the Pytorch and Scikit-learn packages.

#### 2.2.5 Performance metrics

Overall accuracy and confusion matrices were computed to measure the performance of each algorithm with respect to the dataset used. In *Top-N* accuracy, a “correct” prediction denotes a data point whose true class is one of the *N* most probable classes, as predicted by the classification model. For this work, Top-1 accuracy was used. A prediction was considered correct only if the most probable class of a data point matched the true class. For a given class *k*, performance was evaluated using the:


$$\text{Top−}1\text{ accuracy }(\text{class k})=\frac{\;\#\;points\;correctly\;predicted\;as\;class\;k}{\#\;of\;points\;belonging\;to\;class\;k}$$

This evaluation helped identify the strengths of the models discussed, as well as certain classes in which the prediction capabilities of each model should be improved.

Results are presented through confusion matrices. A confusion matrix displays predicted class labels on the horizontal axis and true class labels on the vertical axis, so that the value in the *i^th^* column and the *j^th^* row represents the proportion of data points that belong to class *j* which the model predicted to belong to class *i.* For this reason, the main diagonal of a confusion matrix represents the proportion of each class that is correctly predicted, while every value which is not on the main diagonal represents a proportion of data points in each class that is misclassified by the respective model. For binary classification, the other values represent the false positives (data predicted infection while no infection is detected) and the false negatives (data predicted non-infection while infection is detected). In the multiclass classification, when looking at the non-infected row, the sum of all values that are not correctly predicted are false positives.

To understand how much the predictive power of the CNN and RF models was affected by variability in virus symptom expression at the leaf level, we calculated accuracy by imaging two leaves collected from the same vine and combining them in one single molecular test. When the model classified one leaf as not infected and the other leaf as infected, the “healthy” leaf was reclassified to match the category of the leaf predicted to be infected. Accuracy was then calculated in the same manner as previously done, with the adjusted array of category predictions. In this analysis, a positive difference indicates an increase in accuracy compared to the original accuracy figures. This work was conducted on the whole dataset (including vines sampled in early disease stages) to validate further the challenge posed to the models by the image dataset regarding more variable foliar virus symptoms earlier in the season.

#### 2.2.6 Variable importance rate

For the random forest model, each fold of the cross-validation returned variable importance rankings. For each of the variables (in this case, the different bands of light whose images were used for classification purposes), a variable importance ranking assessed the level of contribution each respective attribute makes to the random forest model. These rankings assessed the strength of the relationships between light bands and prediction accuracy and helped investigate the relationships between each wavelength and the outcome used by the model for prediction (Kuhn & Johnson, 2016).

## 3 Results

### 3.1 Dataset and image segmentation

Leaf samples were collected around veraison (August) when most leafroll-infected and red blotch-infected vines were asymptomatic. Leaf samples were also collected in late September when disease symptoms were more apparent. In total we collected 496 images from 248 plants. From this dataset, two smaller datasets of 319 and 312 images were obtained by including only the pre-symptomatic leaves from August or exclusively the symptomatic leaves from September together with the images of the not-infected leaves. For all samples, the viral infection status was determined by PCR-based tests. Molecular assays revealed the predominance of GRBV and GLRaV-3 in the samples tested by PCR with a few petioles testing positive for GLRaV-1 and GLRaV-2, and many samples testing negative for red blotch and leafroll-associated viruses. For the purpose of this study, individual viruses associated with leafroll were not distinguished; instead, a sample was considered infected by leafroll if it tested positive for one of the four leafroll viruses assayed for in this study.

In all datasets, roughly 1/3 of the images were from leaves collected from non-infected vines and 2/3 were from images of leaves collected from infected vines. The dataset was divided into categories used in the machine-learning models (**Table 1**). Non-infected versus infected categories were used for the binary classification models, and classes 0-3 were used in the multiclassification models.

**Table 1:** Number of images by category according to the dataset used.

Category	Description	Entire dataset	Pre-symptomatic	Symptomatic
0	Non-infected	135	135	135
1	Leafroll	156	50	106
2	Red blotch	108	86	22
3	Leafroll and red blotch	97	48	49
Non-infected		135	135	135
Infected		361	184	177
Total		496	319	312

### 3.2 Binary classification

#### 3.2.1 Accuracy in binary classification

The overall accuracies of the RF and CNN binary models were calculated for the three datasets (**Table 2**). In all the cases, the CNN model performed better than the RF model (from 1.4 points to 4.6 points more than the RF). With the entire dataset, the overall accuracy was 79.5% for the RF model and 80.9% for the CNN model. Using only the pre-symptomatic dataset the overall accuracy increased to 82.8% for RF and 85.6% for the CNN model (**Table 2**). For the symptomatic dataset, the overall accuracy was 82.4% for the RF model and 87% for the CNN model. The highest accuracy was obtain using the CNN model with the symptomatic dataset.

**Table 2:** Overall accuracy of binary classifications for each model and each dataset.

Overall accuracy	CNN model	RF model
Entire dataset	80.9	79.5
Pre-symptomatic dataset	85.6	82.8
Symptomatic dataset	87	82.4

Results are expressed in % points.

**Figure 4:**
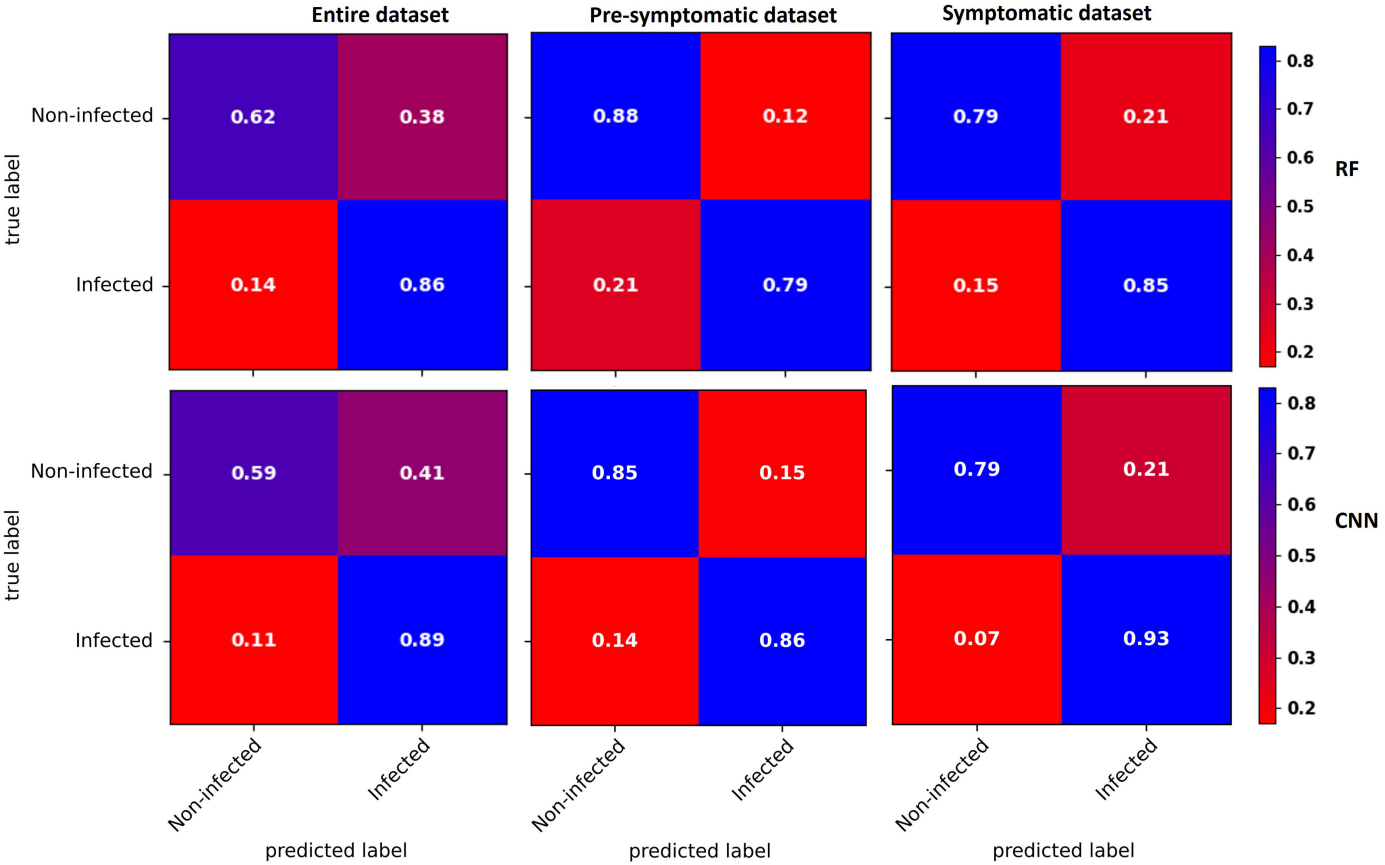
Confusion matrices of binary classification using the RF (top) and CNN (bottom) models with the entire dataset (left), the pre-symptomatic dataset (center) and the symptomatic dataset (right). The top left corner shows the percentage of non-infected vines that were well predicted as non-infected. The top right corner shows the percentage of non-infected vines that were wrongly predicted as infected (false positive). The bottom left corner shows the percentage of infected vines that were wrongly predicted as non-infected (false negative). The bottom right corner shows the percentage of infected vines that were well predicted as infected. Using the pre-symptomatic dataset shows fewer false positives and using the symptomatic dataset shows fewer false negatives and false positives.

Confusion matrices for the three datasets were obtained with the RF and CNN models (**Figure 4**). For the entire dataset, errors were mostly related to false positives, where the CNN model (41% of the non-infected) did slightly worse than the RF model (38% of the non-infected). In contrast, the CNN model did better than the RF model on the false-negative rate (**Figure 4**).

With the pre-symptomatic dataset, the greatest improvements were for the false positives that decreased to more than half in both the RF (38% to 12% of the non-infected) and CNN (41% to 15% of the non-infected) models, resulting in an improved ability to accurately predict non-infected cases from 62% to 88% for the RF model and from 59% to 85% for the CNN model. The false negative rate slightly increased from 14% to 21% for the RF model and from 11% to 14% with the CNN model (**Figure 4**).

For the symptomatic dataset, both the false negative and false positive rates tended to decrease, although the improvement in the false positive was less striking than with the pre-symptomatic dataset (**Figure 4**). The CNN model shows fewer false negatives (7%), improving the prediction accuracy of the infected class to 93%.

#### 3.2.2 Effect of variability in symptom expression at leaf level

The binary accuracies of the original models used to predict infection of each leaf separately and the new accuracies computed combining the prediction of both leaves of the same vine was compared (**Table 3**). When the model classified one leaf as not infected and the other leaf as infected, both leaves were considered as infected, and consequently the whole plant was considered as infected. For both models, the overall accuracy remained almost the same while the accuracy of the infected class improved by 5 points and that of the non-infected decreased by 8 to 11 points. The decrease in non-infected class accuracy can be due to the impact of false positives within the dataset. If the vine was not infected, the two leaves classified as infected corresponded to false positives, and this adjustment reduced the number of correctly predicted non-infected leaves.

**Table 3:** Accuracy comparison of the binary classification models with two-leaf adjusted model prediction scheme on the full dataset. Results are shown in % points.

	RF			CNN		
	Original, single leaf accuracy	Two-leaf adjusted prediction accuracy	Difference	Original, single leaf accuracy	Two-leaf adjusted prediction accuracy	Difference
Overall	79.5	79.7	+0.2	80.9	81.6	+0.7
Non-infected	62	54	-8	59	48	-11
Infected	86	91	+5	89	94	+5

### 3.3 Performance evaluation and exploration of the four-category classification

#### 3.3.1 Accuracy in multiclassification models

Multiclassification models were used to predict infection status in four categories, i.e., non-infected, infected with leafroll only, infected with red blotch only, and co-infected with both viruses, to determine the accuracy for each category with the RF and CNN models using the entire dataset, the pre-symptomatic dataset, or the symptomatic dataset (**Table 4**).

**Table 4:** Accuracy of the RF and CNN models for each dataset and category.

Dataset	Overall	Non-infected	Leafroll	Red blotch	Both
RF					
**Entire dataset**	62.2	67	74	35	67
**Pre-symptomatic**	***77.7***	***90***	81	***46***	***97***
**Symptomatic**	65.7	75	***84***	8	22
CNN					
**Entire dataset**	67	60	86	49	66
**Pre-symptomatic**	***76.9***	79	***92***	53	***98***
**Symptomatic**	73.2	***84***	80	***57***	37

Results are shown in % points and best results are highlighted in italics-bold.

The overall accuracy of the RF model was 62.2% for the entire dataset, and 67% for the CNN model. This accuracy for the pre-symptomatic dataset increased to 77.7% for the RF model and 76.9% for the CNN model. Symptomatic overall accuracy was lower with 65.7% for the RF model and 73.2% for the CNN model. The highest overall accuracy was observed for the CNN model with a difference of 0.8 to 7.5% according to the dataset used (**Table 4**).

Considering performances on predicting individual categories, the results were closer between the two models when using the entire dataset but differences in accuracy increased for the symptomatic dataset (**Table 4**). On this dataset, the largest improvement in accuracy was obtained with the CNN model with respect to the RF model in the red blotch category (+49%), and both virus category (+15%). However, this increase was obtained at the expense of longer training times. Besides training time, there were no significant differences in the prediction time of new samples’ infection status (in the model application).

**Figure 5:**
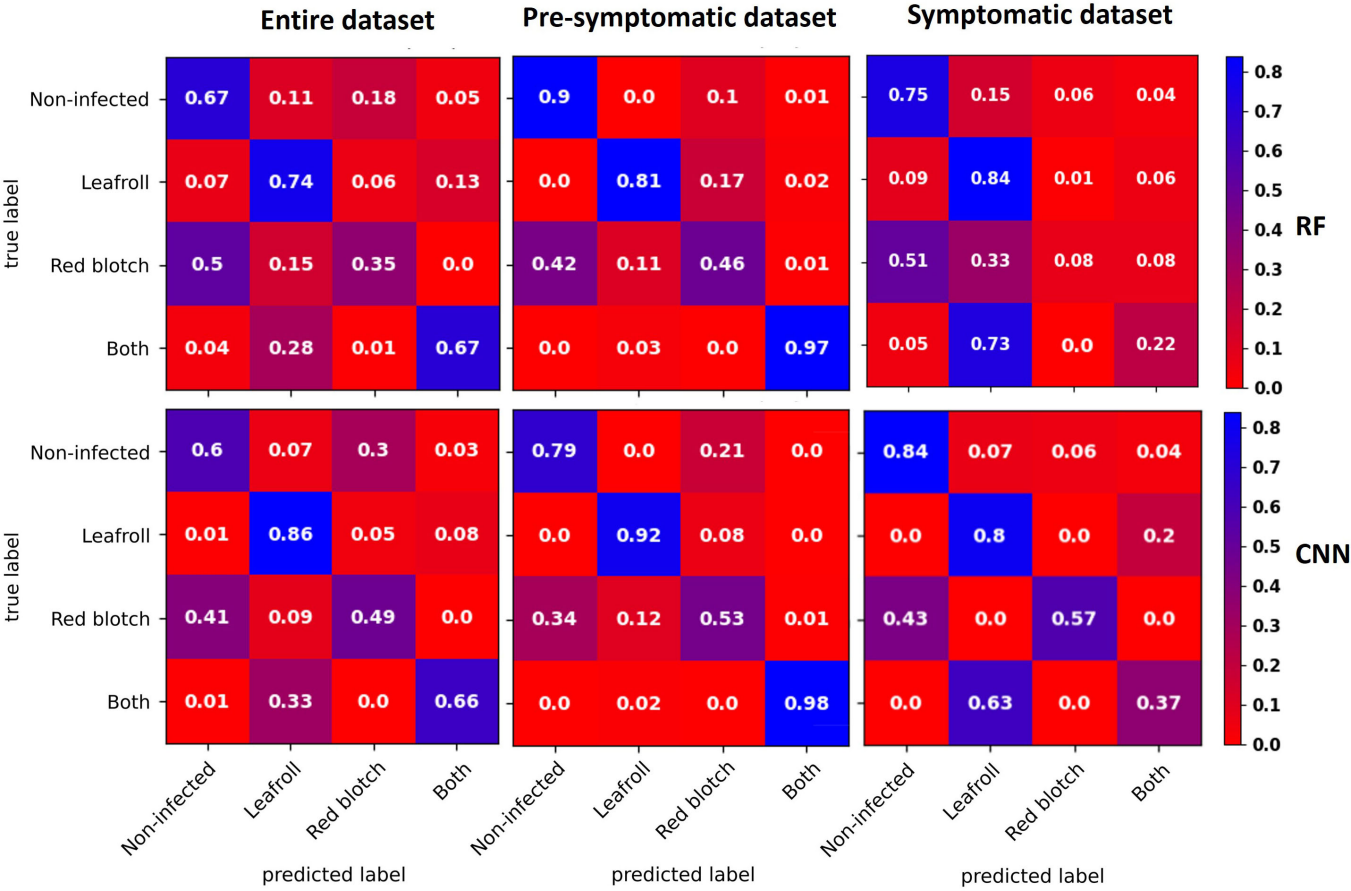
Confusion matrices averaged across the five CV folds for data with the entire dataset (left), the pre-symptomatic dataset (center), and the symptomatic dataset (right) using the RF (top) and CNN (bottom) models. The diagonal represents the percentage for each category that was well predicted. The three percentages below the top left corner represent the false negatives for each infected category. Two categories, non-infected and leafroll, were best predicted by both types of models. Leafroll was the best-predicted class with a maximum of 9% of false negatives using the RF model for the symptomatic dataset.

Confusion matrices for each dataset with the RF and CNN models were used for prediction of each category (**Figure 5**). Two categories, non-infected and leafroll, were best predicted by both types of models. Leafroll was the best predicted class with a maximum of 9% of false negatives using the RF model.

With the full dataset, some GRBV-infected leaves were predicted as non-infected (50% with the RF model and 41% with the CNN model), and one third of the leaves infected with both viruses were predicted as leafroll infected (28% with the RF model and 33% with the CNN model).

Using the pre-symptomatic dataset, GRBV-infected leaves were wrongly predicted as non-infected for 42% by RF and 34% by CNN. Leaves infected with both viruses were well predicted for 97% by RF and 98% by CNN.

For the symptomatic dataset, GRBV-infected leaves were also wrongly predicted as non-infected (51% with the RF model and 43% with the CNN model). The CNN model correctly classified 57% of GRBV-infected leaves, while the RF model mostly predicted them as non-infected (51%) or leafroll infected (33%). Leaves infected by both viruses were mainly predicted as leafroll infected (73% with the RF model and 63% with the CNN model).

#### 3.3.2 Effect of variability in symptom expression at the leaf level

Multiclass accuracies of the original models used to predict infection of each leaf separately and the new accuracies computed using the combined prediction of both leaves of the same vine were compared (**Table 5**). The impact of the two-leaf adjustment method on model performances surpassed what was observed in the binary classification models (**Table 3**). Accuracy improved in the red blotch and both-viruses categories for the CNN and RF models. The largest impact was in the prediction of red blotch-infected samples, with a 14.1% increase in accuracy with the RF model and 10.3 with the CNN model. The CNN model agreed with itself in predicting leaves of the same vine 70% of the time, in contrast to 55% of the time for the RF model. The accuracy of the non-infected category decreased for both models, which is likely due to the impact of false positives within the dataset, as also observed when applied to the binary classification scheme (**Table 3**).

**Table 5:** Accuracy comparison of the multiclassification scheme with two-leaf adjusted model prediction scheme on the full dataset.

	RF			CNN		
	Original single leaf accuracy	Two-leaf adjusted prediction accuracy	Difference	Original single leaf accuracy	Two-leaf adjusted prediction accuracy	Difference
Overall	62.6	62.8	+0.2	67	66	-1
Non-infected	67	55.6	-11.4	60	51.9	-8.1
Leafroll	74	73.7	-0.3	86	81.4	-4.6
Red blotch	35	49.1	+14.1	49	59.3	+10.3
Both	67	69.1	+2.1	66	67	+1

Results are shown in % points.

#### 3.3.3 Variable importance rate of RF

**Figure 6:**
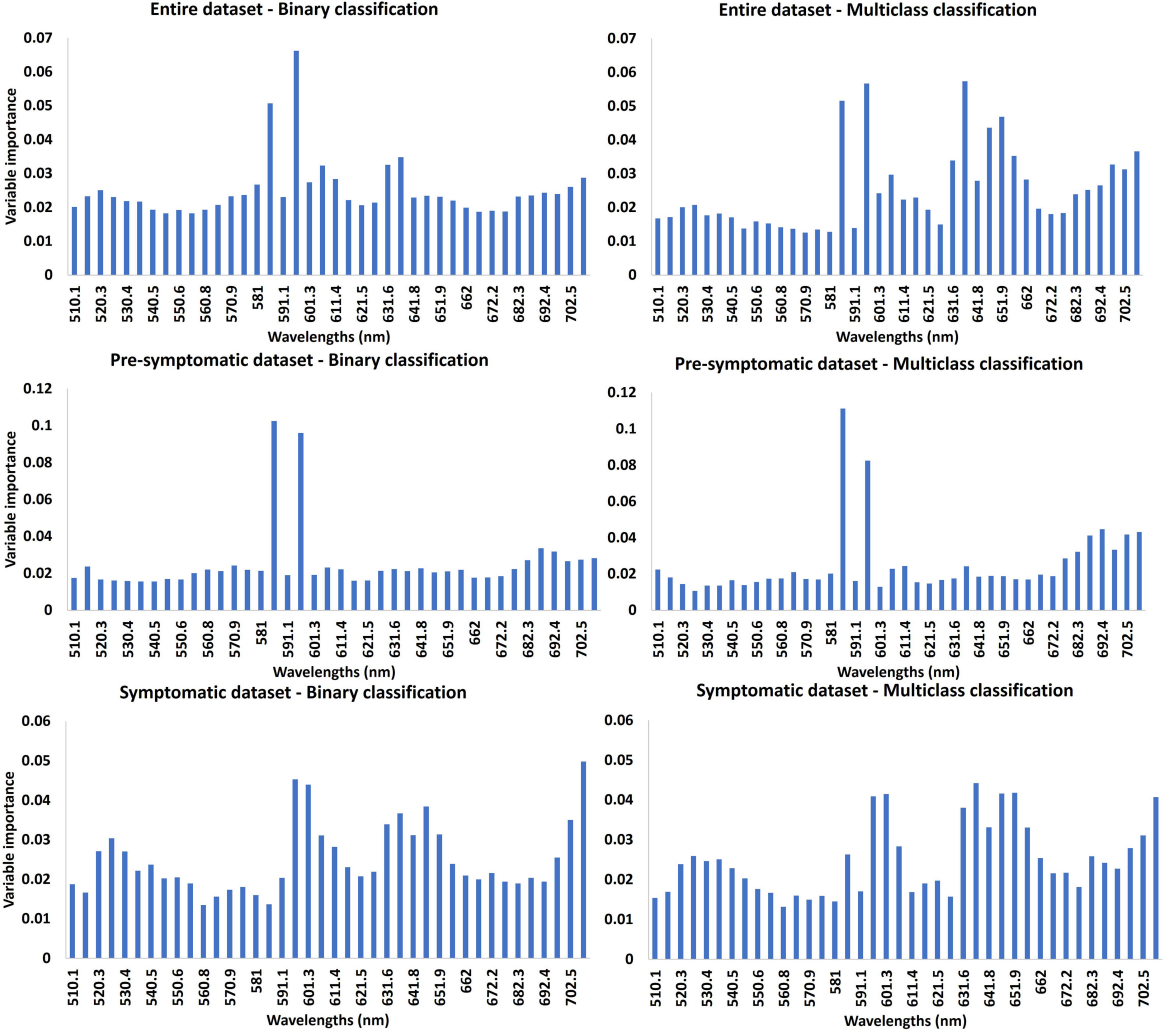
Variable importance (VI) of RF model. The higher the importance of the band, the higher the contribution of the reflectance of this band to the model. The first line represents the VI using the entire dataset, the second line is for the pre-symptomatic dataset and the third line is for the symptomatic dataset. The first column represents the VI for the binary classifications. The second column represents the VI for the multiclass classifications.

The relative importance of each band in terms of contribution to the RF model was analyzed for all the different datasets with binary and multiclass classifications (**Figure 6**). In all cases, a larger number of wavelengths was relatively more important for multiclass classifications than for binary classifications. More wavelengths were also highlighted for the classifications with the symptomatic dataset compared with the pre-symptomatic dataset. For the pre-symptomatic dataset with both binary and multiclass classifications, two wavelengths appeared to be dominant at 586 nm and 596 nm in the yellow region. Concerning the symptomatic dataset, the important wavelengths belong to the green (~530 nm), orange (~600 nm), red (650 nm), and the beginning of the red-edge regions (~700 nm).

### 3.4 Visual assessment

The difficulty in visually assessing virus infections on our datasets was confirmed by the performance of expert predictions using RGB segmented images (**Table 6**). The binary classification accuracy was about 50% independent of the dataset. Accuracy improved in the four-category classification to 40% overall (selecting the right category by chance would be 25%).

**Table 6:** Overall accuracy of human prediction for binary and multi-category classification with full or reduced dataset.

	Binary	Multiclass
Dataset with early symptoms	50.23	39.77
Dataset without early symptoms	50.80	41.93

Results are shown in %.

## 4 Discussion

### 4.1 Model performances and comparison

#### 4.1.1 Comparing the accuracy of random forest with convolutional neural networks

This study tested two machine learning algorithms, a tree-based ensemble learning method (RF) and a deep neural network method (CNN). The CNN model outperformed the RF model in most of the cases by up to 7.5 points with regards to the overall accuracy for the four-category classification (**Table 4**, **Figure 5**) and up to 4.6 points with regards to the binary classification (**Table 2**). This is in accordance with previous studies comparing the CNN with RF models for land classification (Jozdani et al., 2019; Yoo et al., 2019) and recently for plant disease detection (Hatuwal et al., 2020; Musci et al., 2020).

For the binary classifications, the false negative rate decreased with the use of the CNN compared to the RF model. This can be explained by the fact that we had to train the RF algorithm on the average spectral signal and the CNN model on the whole hypercube.

#### 4.1.2 Comparison of model performances with the literature

The non-infected and leafroll-infected grapevine leaves were the best predicted categories (up to 86% for leafroll prediction with the CNN model using the entire dataset and up to 92% with the pre-symptomatic dataset). These categories were also the ones with the largest number of samples in most of the cases, which may affect the model performances. Similarly, Naidu et al. (2009) obtained 81% accuracy using spectral indices computed with data from a visible-near (350-2500nm) portable spectrometer to classify leafroll-infected vs. non-infected leaves with both symptomatic and non-symptomatic data. In our study, the capability of a hyperspectral camera was leveraged, thus maintaining the spatial information used in the CNN model, but the spectral region was limited to the visible. This was to avoid possible changes in the spectrum in the near infrared region due to changes in water content with leaf storage, for example, that could have affected the results. Other studies used all the spectral information contained in hyperspectral images from 500 to 2500nm to predict leafroll infection and obtained a classification accuracy exceeding 90%, mostly using leaves or plants with fully expressed symptoms (MacDonald et al., 2016; Sinha et al., 2019; Bendel et al., 2020). However, these studies used a binary classification where plants infected with a single virus (mostly leafroll) were differentiated from non-infected vines but did not attempt a multiclass approach, neither included multiple virus symptoms for a binary classification. In our study, binary classification reached greater accuracy (87% overall accuracy of the CNN model with the symptomatic dataset), despite the complexity related to the presence of multiple viruses. In California, there may not be the need to differentiate between leafroll and red blotch viruses in a vineyard context, as the final decision (i.e., removing infected vines) would be the same, regardless of the virus, and a binary classification offering higher accuracy and lower false negatives would be appropriate. In other regions, where GRBV vector is absent, a binary model for the detection of GLRaV is sufficient.

#### 4.1.3 Comparison with visual assessment

As highlighted by Cruz et al. (2019), few studies on disease detection using artificial intelligence compare their performance with visual assessment, albeit being important to discuss the potential of machine learning algorithms. Here we did not accomplish a rigorous assessment and do not wish to claim the superiority of hyperspectral imaging over visual identification. Our intent was to characterize how challenging it was to predict this dataset (because oftentimes symptoms were not visible in infected vines) and we used visual assessment as the reference. In our example, accurately differentiating leafroll or red blotch-infected leaves was very challenging using RGB segmented images for our two experts (**Table 6**). With an overall accuracy of up to 87% with a binary classification, machine learning models could help identify vines and increase screening speed. Although comparative performance analyses with visual observation should be performed in the vineyard on full grapevine with more experts, as high accuracy rates can be achieved *in-situ* by experienced personnel (Bell et al., 2017).

### 4.2 Effect of different parameters on model performances

#### 4.2.1 Effect of symptom variability

Grapevine virus disease symptoms vary during the growing season (Poojari et al., 2017; Rumbaugh et al., 2021). As our field campaign took place during several plant development stages, infected vines did not show the same severity of foliar symptoms. For the binary classification, the false negative rate was lower using the symptomatic dataset compared with the pre-symptomatic dataset. This can be explained by the absence or low level of symptoms of infected vines from the earlier dataset despite some wavelengths in the yellow domain seeming to be informative (**Figure 6**).

According to the confusion matrices computed for the RF and CNN models, leaves infected with GRBV were predicted as non-infected (from 34% to 51%). This might be explained by the symptom expression level of red blotch leaves used in this study. Indeed, the red blotch symptoms were observed to be overall milder than leafroll symptoms. Symptoms on leaves infected only by GRBV were often confused with healthy leaves, and leaves infected with both viruses showed typical leafroll symptoms and were sometimes predicted to be infected with only leafroll viruses. The only study using hyperspectral imaging to detect GRBV demonstrated the possibility of separating the parts of the leaves with or without symptoms using a Support Vector Machine (SVM) classifier (Mehrubeoglu et al., 2016). As this virus has been less investigated because of its more recent discovery (Sudarshana et al., 2015), further studies are needed to evaluate the potential of hyperspectral images to detect it. To our knowledge, our study is the first to identify leaves affected by this virus within a dataset obtained with healthy and leafroll-infected leaves.

The effect of the variability in disease symptom expression on model performances was enhanced when working on a single leaf rather than two leaves per plant as the basis for model development. Combining the prediction on two leaves from the same plant substantially increased the accuracy of the red-blotch category. This is because when symptoms are variable or not strongly expressed, the rate of false negatives is greater than the rate of false positives. Therefore, even though our strategy increased the number of false positive classifications in most cases, there was a reduction of false negatives. This improvement is beneficial in a vineyard setting where false negatives represent infected plants that are not detected, and thus may contribute to virus spread by insect vectors to healthy plants until correctly detected and eliminated. As described by AL-Saddik et al. (2017) and Boulent et al. (2020), an incorrect negative prediction that keeps an infected plant in place is far more costly than a false positive prediction, leading to the removal of a healthy plant.

#### 4.2.2 Effect of the number of samples

We noticed that the overall accuracies using the symptomatic dataset are lower than using the pre-symptomatic dataset in most of the cases (-0.2 to –12 points), except for the binary classification using the CNN (+1.4 points). This may mostly be due to the poor accuracy of the red blotch-infected category for which the number of samples is considerably lower for the symptomatic dataset (22 samples) than for the pre-symptomatic dataset (86 samples). This observation is even more noticeable for the multiclass classification using the RF model. In this case, efforts to balance the dataset by lowering the number of non-infected and leafroll categories, the accuracy of the red blotch category reached 27% (4% with the original dataset), but the accuracy of the two other classes decreased (**Supplementary Figure 2**). In our dataset, two different wine grape cultivars were mixed with the intent of training a model that could learn general features of virus symptoms and eventually generalize infection. A sideback benefit of this approach is that the difference between cultivars could be an additional piece of information for the model (Gutiérrez et al., 2018). Future developments of this work should focus on increasing the number of leaves imaged and trying to have a well-balanced dataset for each category. This might prove challenging because the composition of the dataset for each category can only be ascertained after the images are taken and the virus diagnostic tests are complete.

### 4.3 Spectral domains used

This study was performed using wavelengths from 510 nm to 710nm. These wavelengths belong to the visible domain that enables the assessment of pigment content (Hodáňová, 1985; Carter and Knapp, 2001). The variable importance rate computed with the RF model highlighted the most important bands, which were mainly located in the yellow region for the pre-symptomatic dataset and in the green, orange, and red sub-regions for the symptomatic dataset. This can be explained by the color change caused by both diseases after veraison: symptomatic leaves turn from green to red in red-berried wine grape cultivars such as Cabernet franc and Cabernet Sauvignon. The green color is due to the chlorophyll content (Main et al., 2011; Behmann et al., 2014; Matese and Di Gennaro, 2015), while the red is due to the increase of anthocyanins in leaves (Gamon and Surfus, 1999), as a response to pathogen attack (Himeno et al., 2014).

As demonstrated by Martínez-Lüscher et al. (2019), GRBV causes a reduction in photosynthesis which may have an impact on chlorophyll and carotenoid concentration. The same observation was made for GLRaV (Endeshaw et al., 2014). Such findings are consistent with the wavelengths identified as most important for the RF model with the symptomatic dataset (**Figure 6**). These wavelengths are close to the regions of maximum absorption of chlorophyll a and b, 642 nm and 626 nm, respectively. The visible domain is of interest to assess pigment concentration, and the accuracy obtained in this study is promising to identify healthy and leafroll-infected plants. Further investigations could focus on the use of more spectral domains in the near-infrared and shortwave infrared spectral regions for the detection of asymptomatic, virus-infected grapevines (Nguyen et al., 2021). Indeed, these domains can reflect the cellular structure or leaf water content which can be affected by diseases (Junges et al., 2020). In that case, it will be important to work with images of whole canopies instead of detached leaves.

## 5 Conclusion

Grapevine leafroll-associated viruses and grapevine red blotch virus negatively impact vineyard health and wine quality. There is no cure for these two viruses in the vineyard. The only way to limit their secondary spread is to identify infected plants, remove them, and replace them with clean plants. Identifying virus symptoms in the vineyard for removal (aka roguing) is time-consuming and costly. A rapid decision tool would be beneficial to the grape and wine industries to deal with this challenge. In this study, hyperspectral images were used for the identification of both groups of viruses using two different machine learning models (CNN and RF) on pre- and symptomatic datasets. The best results were obtained using a CNN model with a dataset where samples from infected vines were acquired at the time when symptoms were more apparent (87% overall accuracy with a binary classification on a symptomatic dataset) or when the model used two leaves rather than a single leaf per vine. Therefore, working with a larger number of leaves per plant and utilizing the most balanced dataset possible (number of samples per category) is recommended when assessing virus infection from hyperspectral images in the laboratory.

This study investigated for the first time a multiclassification distinguishing non-infected grapevine leaves, leaves infected with GRLaV, leaves infected with GRBV, or those co-infected with both viruses. This was challenging both from machine learning and from visual assessment standpoints, though our preliminary results are promising. Further investigations are needed to increase prediction performances, especially for the detection of GRBV-infected plants with an extended number of samples. This work focused on the visible region of the light spectrum. Within this range, the most informative wavelengths to predict virus presence were in the red and orange regions (anthocyanins) or associated with chlorophyll and carotenoid absorption. Extending to a larger region of the electromagnetic spectrum will be important when assessing difficult to classify vines. Finally, a scale change, i.e., leaf versus canopy, can significantly improve developing an operational tool to detect diseases in grapevines. Further work will be need to treat images acquired over whole vines from the ground or the air as a basis for future studies of virus detection in vineyards using hyperspectral imaging.

## Data availability statement

The raw data supporting the conclusions of this article will be made available by the authors, without undue reservation.

## Author contributions

LB, MC, and MFu contributed to conception and design of the study and secured fundings. LB, and MFu lead and contributed to the data collection, MC and MFl contributed to the data collection. ES organized the database. ES, BC, EL-P and LB performed the statistical analysis. ES and EL-P wrote the first draft of the manuscript. LB and MFu contributed to the writing. All authors contributed to the article and approved the submitted version.
