# Supplementary material for: Phenotyping grapevine red blotch virus and grapevine leafroll-associated viruses before and after symptom expression through machine-learning analysis of hyperspectral images
Source: Front Plant Sci. 2023 Mar 10;14:1117869. doi: 10.3389/fpls.2023.1117869 (PMC10036814; doi:10.3389/fpls.2023.1117869)
Supplement: Supplementary file 1 [file DataSheet_1.docx]

Supplementary Material

**Phenotyping grapevine red blotch virus and grapevine leafroll-associated viruses before and after symptom expression through machine-learning analysis of hyperspectral images**

**E. Sawyer^1,2+^, E. Laroche-Pinel^1+^, Flasco M.^3^, Cooper M.L.^4^, Corrales B.^1^, Fuchs M.^3^, Brillante L.^1^***

*** Correspondence:** Luca Brillante: lucabrillante@csufresno.edu

# Supplementary Figures


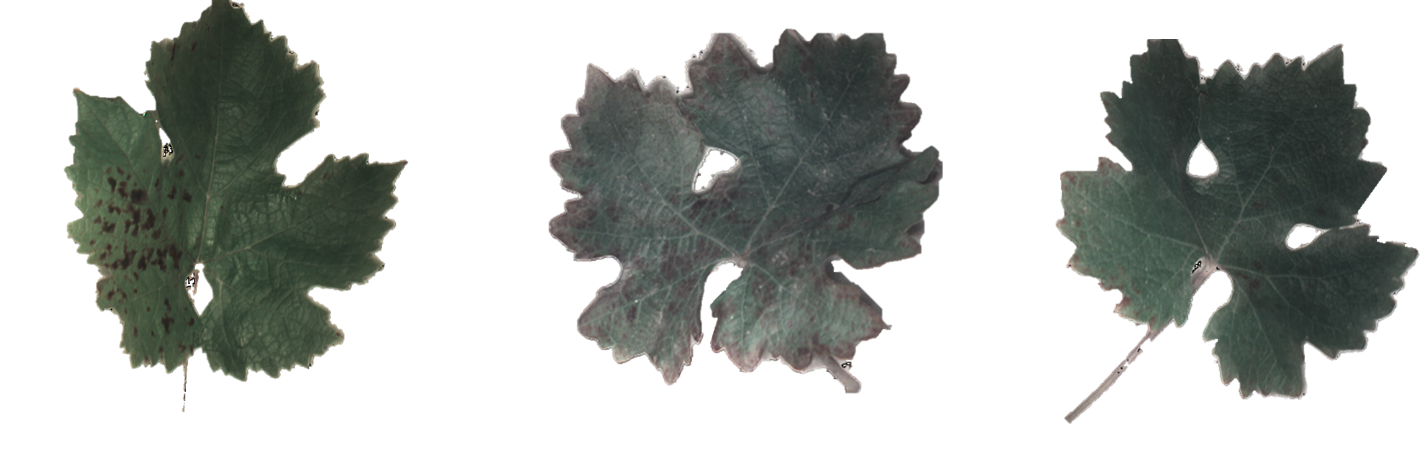


**Supplementary Figure 1.** Example of grapevine leaves in the dataset showing reddening symptoms but testing negative for GRLaVs and GRBV in PCR.


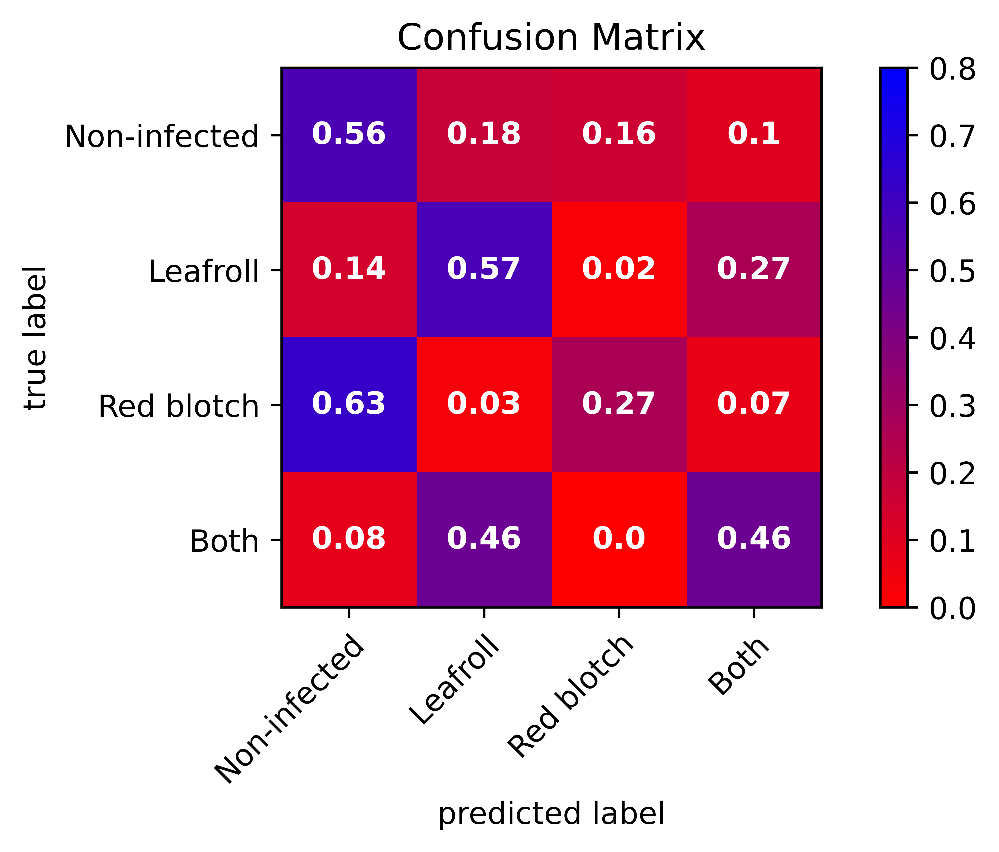


**Supplementary Figure 2.** Confusion matrix obtained with the RF model using the symptomatic dataset with a reduced number of non-infected and leafroll-infected leaves to test a more balanced dataset (Non-infected: 55, leafroll-infected: 56, red blotch-infected: 22, co-infected: 49).
